# Combination Therapy with a Sodium-Glucose Cotransporter 2 Inhibitor and a Dipeptidyl Peptidase-4 Inhibitor Additively Suppresses Macrophage Foam Cell Formation and Atherosclerosis in Diabetic Mice

**DOI:** 10.1155/2017/1365209

**Published:** 2017-03-19

**Authors:** Michishige Terasaki, Munenori Hiromura, Yusaku Mori, Kyoko Kohashi, Hideki Kushima, Makoto Ohara, Takuya Watanabe, Olov Andersson, Tsutomu Hirano

**Affiliations:** ^1^Department of Medicine, Division of Diabetes, Metabolism, and Endocrinology, Showa University School of Medicine, Tokyo, Japan; ^2^Department of Cell and Molecular Biology, Karolinska Institutet, Stockholm, Sweden; ^3^Laboratory of Cardiovascular Medicine, Tokyo University of Pharmacy and Life Sciences, Tokyo, Japan

## Abstract

Dipeptidyl peptidase-4 inhibitors (DPP-4is), in addition to their antihyperglycemic roles, have antiatherosclerotic effects. We reported that sodium-glucose cotransporter 2 inhibitors (SGLT2is) suppress atherosclerosis in a glucose-dependent manner in diabetic mice. Here, we investigated the effects of combination therapy with SGLT2i and DPP-4i on atherosclerosis in diabetic mice. SGLT2i (ipragliflozin, 1.0 mg/kg/day) and DPP-4i (alogliptin, 8.0 mg/kg/day), either alone or in combination, were administered to *db/db* mice or streptozotocin-induced diabetic apolipoprotein E-null (*Apoe^−/−^*) mice. Ipragliflozin and alogliptin monotherapies improved glucose intolerance; however, combination therapy did not show further improvement. The foam cell formation of peritoneal macrophages was suppressed by both the ipragliflozin and alogliptin monotherapies and was further enhanced by combination therapy. Although foam cell formation was closely associated with HbA1c levels in all groups, DPP-4i alone or the combination group showed further suppression of foam cell formation compared with the control or SGLT2i group at corresponding HbA1c levels. Both ipragliflozin and alogliptin monotherapies decreased scavenger receptors and increased cholesterol efflux regulatory genes in peritoneal macrophages, and combination therapy showed additive changes. In diabetic *Apoe^−/−^* mice, combination therapy showed the greatest suppression of plaque volume in the aortic root. In conclusion, combination therapy with SGLT2i and DPP4i synergistically suppresses macrophage foam cell formation and atherosclerosis in diabetic mice.

## 1. Introduction

Dipeptidyl peptidase-4 inhibitors (DPP-4is) and sodium-glucose cotransporter 2 inhibitors (SGLT2is) have been increasingly used for the treatment of type 2 diabetes. Several preclinical studies [1–4] including ours [5, 6] have demonstrated that DPP-4is confer antiatherosclerotic effects independent of antihyperglycemic actions. In addition, we recently reported that SGLT2i suppresses the acceleration of atherosclerosis in diabetic apolipoprotein E-null (*Apoe^−/−^*) mice and macrophage foam cell formation in diabetic *db/db* mice in a glucose-dependent manner [7]. These findings raised the possibility that combination therapy with SGLT2i and DPP-4i could synergistically further suppress atherosclerosis and macrophage foam cell formation by both glucose-dependent and glucose-independent mechanisms.

Subendothelial accumulation of lipid laden macrophage-derived foam cells occurs during the early stage of atherosclerosis [8]. Accumulation of cholesterol esters in macrophages is a hallmark of foam cell formation that depends on the uptake of oxidized low-density lipoprotein (ox-LDL) via its receptors, lectin-like ox-LDL receptor-1 (Lox-1) and CD36, and on the efflux of free cholesterol by ATP-binding cassette transporter A1 (ABCA1) and ATP-binding cassette subfamily G member 1 (ABCG1) [8].

In the present study, we aimed to evaluate whether combination therapy with SGLT2i and DPP-4i was superior to monotherapy with either an inhibitor alone in suppressing macrophage foam cell formation, a critical process in atherosclerosis, or the expression of genes involved in foam cell formation in *db/db* mice, a model of type 2 diabetes. In addition, we evaluated atherosclerotic plaque lesion in streptozotocin-injected *Apoe^−/−^* mice, a model of atherosclerosis exacerbated by hyperglycemia.

## 2. Materials and Methods

### 2.1. Animal Experiment Number 1

Six-week-old male *db/db* mice (a mouse model of type 2 diabetes) were purchased from Sankyo Labo Service (Japan) and kept on standard rodent chow. All the mice showed fasting blood glucose (FBG) levels above 200 mg/dL at the age of 8 weeks. At the age of 9 weeks, we administered a normal diet comprising none, SGLT2i (ipragliflozin, 1.0 mg/kg/day), DPP-4i (alogliptin, 8.0 mg/kg/day), or this combination at these same respective doses to the mice. Ipragliflozin and alogliptin were gifted from Astellas Pharma Inc. (Tokyo, Japan) and Takeda Pharmaceutical Company Limited (Osaka, Japan), respectively. After the treatment period for 4 weeks, peritoneal macrophages were harvested and the mice were sacrificed under general anesthesia with isoflurane [5–7, 9, 10]. All procedures were approved by the Animal Care Committee of Showa University School of Medicine.

### 2.2. Measurements

Blood samples collected after a 6-hour fasting were used for analysis. Hemoglobin A1c (HbA1c) levels were measured using an A1C Now-Plus (Bayer, Frankfurt, Germany) before sacrificing the animals. Plasma levels of glucose, total cholesterol, high-density lipoprotein (HDL) cholesterol, and triglyceride were measured by enzymatic methods (WAKO, Osaka, Japan). Plasma levels of insulin, active glucagon-like peptide-1 (GLP-1), and total glucose-dependent insulinotropic polypeptide (GIP) were determined by enzyme-linked immunosorbent assay (ELISA) (ultrasensitive mouse insulin ELISA kit from MORINAGA, Kanagawa, Japan; GLP-1 (active) ELISA and Rat/Mouse GIP (total) ELISA from EMD Millipore (MA, USA). Blood pressure and pulse were measured on the day of sacrifice in the fasting state using the tail-cuff method (Model MK-2000ST, Muromachi Kikai Co., Ltd., Tokyo, Japan) [5–7, 9, 10].

### 2.3. Oral Glucose Tolerance Tests (OGTTs)

In a subset of animals, OGTTs were conducted as described previously [6, 7]. In brief, glucose (0.5 g/kg body weight) was administered via oral gavage after a 6-hour fast, and the blood glucose levels were measured at 0 (fasting glucose level), 15, 30, 60, and 120 minutes.

### 2.4. Cell Culture

Peritoneal macrophages were collected as described previously [5–7, 9]. After intraperitoneal injection of thioglycolate broth, peritoneal cells were isolated. The cells were seeded onto 3.5 cm dishes (3 × 10^6^ cells/dish) and allowed cell adhesion to the dish for 1 hour of incubation. The adherent cells were identified as peritoneal macrophages and used for a reverse transcription polymerase chain reaction (RT-PCR) or a cholesterol esterification assay [5–7, 9].

### 2.5. Cholesterol Esterification Assay

Adherent macrophages were incubated in the RPMI-1640 medium that contained 10 *μ*g/mL human ox-LDL plus 0.1 mmol/L [^3^H]-oleate conjugated with bovine serum albumin for 18 hours. The cellular lipids were extracted, and the radioactivity of the cholesterol [^3^H]-oleate was determined by thin-layer chromatography [5–7, 9].

### 2.6. Gene Expression

Total RNA was isolated from peritoneal macrophages using QIAGEN reagent (Hilden, Germany). The gene expressions were assessed by real-time RT-PCR using the TaqMan gene expression assay and a sequence detection system (ABI PRISM 7900, Life Technologies, CA, USA) [5–7, 9]. The following primers were used: Lox-1, Mm00454586_m1; CD36, Mm01135198_ml; ABCA1, Mm00442646_ml; ABCG1, Mm00437390_m1; and glyceraldehyde-3-phosphate dehydrogenase (GAPDH), Mm03302249_g1.

### 2.7. Animal Experiment Number 2

Nine-week-old male *Apoe^−/−^* mice (BALB/c background) were purchased from Sankyo Labo Service and kept on a standard rodent chow until the age of 15 weeks. The animals received intraperitoneal injections of streptozotocin to induce diabetes (80 mg/kg/day at 15 weeks old and 50 mg/kg/day at 18 weeks old on each of 5 consecutive days). The fasting blood glucose levels were measured 10 days after the last injection. All the mice showed fasting blood glucose levels higher than 200 mg/dL and were enrolled in this experiment as diabetic mice. At the age of 20 weeks, the diet was switched to an atherogenic diet containing 30% fat, 20% sucrose, 8% NaCl, and 0.15% cholesterol (Oriental Yeast, Tokyo, Japan), and the mice were randomly assigned to treatment with none, SGLT2i (ipragliflozin, 1.0 mg/kg/day), DPP-4i (alogliptin, 8.0 mg/kg/day), or this combination. After 4 weeks of these treatments, the mice were sacrificed under general anesthesia with isoflurane, and the aortas were collected after perfusion fixation. The cross sections taken from the aortic roots were stained with oil red O to evaluate plaque volume or immunostained using antimouse MOMA-2 antibody (1 : 60; Abcam Japan, Tokyo, Japan) to evaluate macrophage infiltration in plaques. The aorta from the ascending aorta to the bifarcation of the common iliac arteries was longitudinally dissected and stained with oil red O. The images were quantified using image analyzers (Adobe Photoshop, San Jose, CA, USA; ImageJ software, Bethesda, MD, USA).

### 2.8. Statistical Analysis

Values are expressed as mean ± SEM. Statistical analysis was performed using analysis of variance (ANOVA) followed by Tukey's post hoc test using JMP software (version 11; SAS Institute Inc., Cary, NC, USA). The correlation between two values was determined using Pearson's correlation test. The significance level was defined as *p* < 0.05.

## 3. Results

### 3.1. Characteristics and Laboratory Data of *db/db* Mice

Table 1 shows the characteristics and laboratory data of diabetic *db/db* mice treated with none, ipragliflozin, alogliptin, and the combination. Mice treated with ipragliflozin or the combination showed higher water intake and urine volume than the others as expected. Ipragliflozin strongly decreased FBG and HbA1c levels, while alogliptin led to modest decreases. Combination therapy did not lead to further decreases in FBG and HbA1c levels compared to ipragliflozin monotherapy. Plasma levels of insulin, active GLP-1, and total GIP significantly increased in mice receiving alogliptin or combination therapy, suggesting effective DPP-4 inhibition. There were no differences in plasma lipid levels between the groups.

### 3.2. Glucose Tolerance

**Figure 1:**
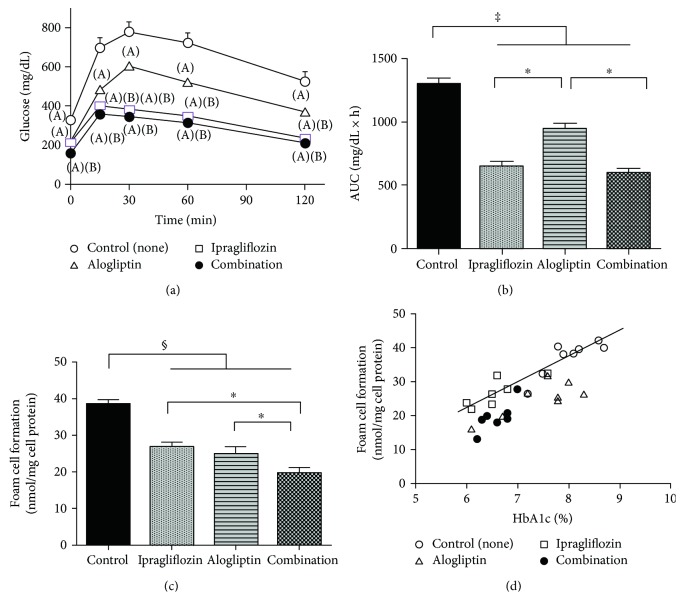
(a) Oral glucose tolerance test in *db/db* mice treated with none, ipragliflozin, alogliptin, ipragliflozin plus alogliptin (combination). *p* < 0.05 versus control (A); *p* < 0.05 versus alogliptin (B). (b) The area under the blood glucose curve for OGTTs. ^∗^*p* < 0.05, ^‡^*p* < 0.001. (c) Foam cell formation of peritoneal macrophages in *db/db* mice.^∗^*p* < 0.05, ^§^*p* < 0.0005. (d) Correlation between macrophage foam cell formation and hemoglobin A1c (HbA1c) levels in *db/db* mice. The line indicates the correlation curves for the control and ipragliflozin groups.

Treatment with ipragliflozin significantly improved glucose loading after OGTT and the AUC, while alogliptin led to only modest amelioration of glucose intolerance. Glucose intolerance was not further suppressed in mice receiving combination therapy compared to ipragliflozin alone consistent with FBG and HbA1c levels (Figures 1(a) and 1(b)).

### 3.3. Foam Cell Formation in Peritoneal Macrophages

The suppression of ox-LDL-induced cholesterol ester accumulation, indicative of the extent of foam cell formation, was comparable between the ipragliflozin and alogliptin monotherapy groups (31% and 35%, resp., Figure 1(c)); both significantly lower than the control group (*p* < 0.0005). Furthermore, the extent of foam cell formation suppression was enhanced by combination therapy (49%), compared to the ipragliflozin and alogliptin monotherapy groups (*p* < 0.05, Figure 1(c)).

### 3.4. Correlation between Macrophage Foam Cell Formation and Glycemic Control

We examined the relationship between macrophage foam cell formation and glycemic control in each animal. The extent of foam cell formation was significantly correlated with HbA1c levels in all treatment groups (control, *r* = 0.76; ipragliflozin, *r* = 0.73; alogliptin, *r* = 0.78; and combination, *r* = 0.8; *p* < 0.05 for all) (Figure 1(d)). The comparison of correlation curves revealed that the extent of foam cell formation was similar between the control and ipragliflozin monotherapy groups at corresponding HbA1c levels. However, the extent of foam cell formation suppression was disproportionately higher in the alogliptin monotherapy or combination therapy group compared to the control and ipragliflozin monotherapy groups at corresponding HbA1c levels (Figure 1(d)). There were no significant correlations between the extent of foam cell formation and body weight, SBP, insulin, total cholesterol, or triglyceride.

### 3.5. Changes in Gene Expression Associated with Foam Cell Formation in Peritoneal Macrophages

**Figure 2:**
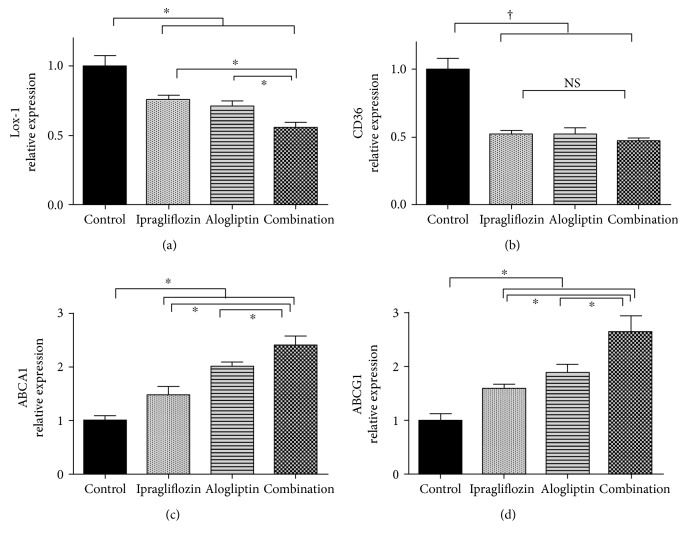
The mRNA levels of Lox-1 (a), CD36 (b), ABCA1 (c), and ABCG1 (d) in peritoneal macrophages isolated from *db/db* mice receiving the indicated treatments. ^∗^*p* < 0.05, ^†^*p* < 0.01.

**Figure 3:**
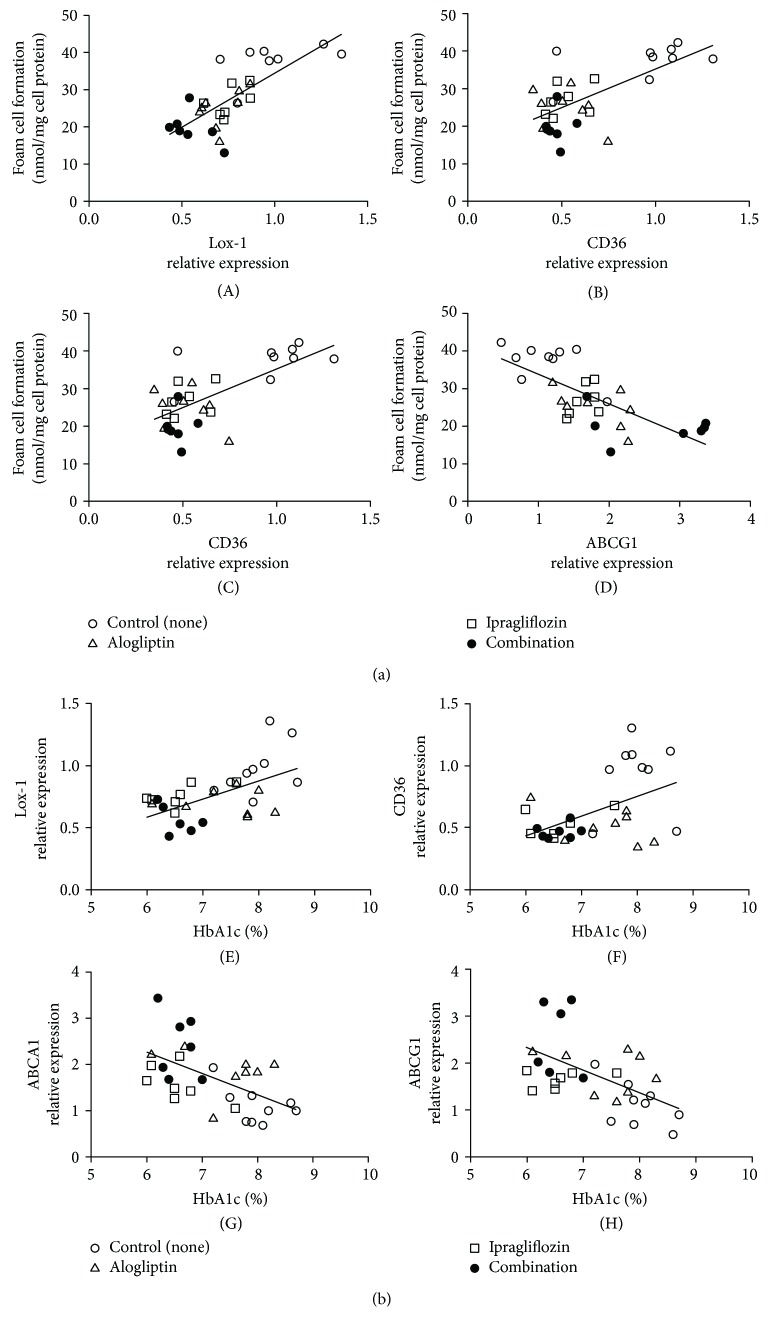
(a) Correlation between foam cell formation and the mRNA levels of genes associated with foam cell formation in peritoneal macrophages isolated from diabetic *db/db* mice. Lox-1, (A); CD36, (B); ABCA1, (C); ABCG1, (D). (b) Correlation between the mRNA levels of genes associated with foam cell formation and HbA1c levels in *db/db* mice. Lox-1, (E); CD36, (F); ABCA1, (G); ABCG1, (H).

There was a significant decrease in Lox-1 expression in both the ipragliflozin and alogliptin monotherapy groups, compared with the control group; Lox-1 expression was further suppressed by combination therapy (Figure 2(a)). The expression of CD36 significantly decreased in the ipragliflozin, alogliptin, and combination groups (Figure 2(b)). Additionally, ABCA1 and ABCG1 mRNA levels were upregulated by treatment with ipragliflozin or alogliptin alone and were further increased by combination therapy (Figures 2(c) and 2(d)). Furthermore, the mRNA levels of both the scavenger receptors, Lox-1 and CD36, and the mediators of cholesterol efflux, ABCA1 and ABCG1, were significantly correlated with foam cell formation (Lox-1, *r* = 0.806; CD36, *r* = 0.675; ABCA1, *r* = −0.798; and ABCG1, *r* = −0.724; Figure 3(a)) and HbA1c levels (Lox-1, *r* = 0.597; CD36, *r* = 0.483; ABCA1, *r* = −0544; and ABCG1, *r* = −0.524; Figure 3(b)), indicating a strong association with the suppression of macrophage foam cell formation and controlling hyperglycemia.

### 3.6. Atherosclerosis in Diabetic *Apoe^−/−^* Mice

**Figure 4:**
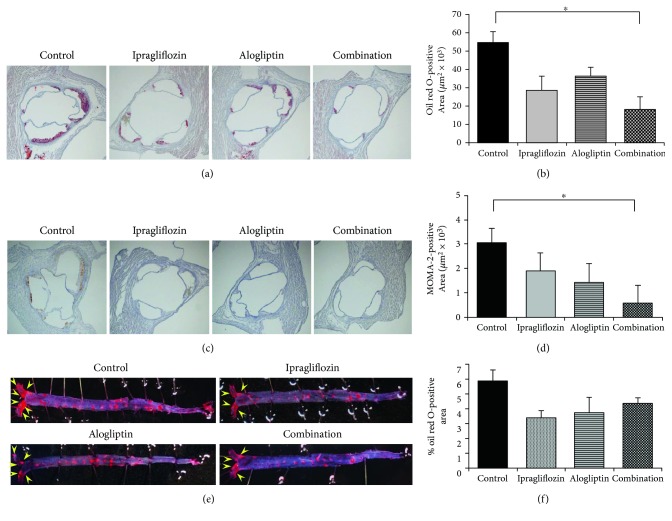
Atherosclerotic plaque formation in streptozotocin-induced diabetic apolipoprotein E-null mice with indicated treatments. (a) Representative images of the aortic root stained with oil red O at 40x magnification. (b) Average oil red O-positive area in the aortic root. (c) Representative images of the aortic root immunostained with antimouse MOMA-2 antibody at 40x magnification. (d) Average MOMA-2-positive area in the aortic root. (e) Representative images of atherosclerotic plaque lesions on the surface of the whole aorta stained with oil red O. The yellow arrows show notable atherosclerotic lesions. (f) Average percentage of oil red O-positive area to the total area of the aorta. ^∗^*p* < 0.05.

Following the confirmation of the additive antiatherosclerotic effects of combination therapy in *db/db* mice, we also evaluated its efficacy on atherosclerosis in diabetic *Apoe^−/−^* mice. The physiological and biochemical data are shown in Table 2. Daily food intake was significantly higher in the combination therapy group than the other groups, and pulse rate was lower in the alogliptin group than the control and ipragliflozin groups. Treatment with ipragliflozin alone and the combination significantly reduced HbA1c levels compared with the control. The other parameters had no significant differences between the groups. Although ipragliflozin and alogliptin monotherapies tended to reduce atheromatous plaque volume in the aortic root, these changes did not reach statistical significance (*p* = 0.1–0.2). However, combination therapy significantly reduced atheromatous plaque volume by 67% compared to the control (Figures 4(a) and 4(b)). In addition, combination therapy showed the greatest suppression of intraplaque macrophage infiltration assessed by MOMA-2-positive area (Figures 4(c) and 4(d)). Atherosclerotic plaque formation on the surface of the whole aorta tended to be suppressed by ipragliflozin, alogliptin, or the combination; however, their suppressions had no significant differences (Figures 4(e) and 4(f)).

## 4. Discussion

The present study is the first to demonstrate that combination therapy with SGLT2i and DPP-4i prevents macrophage foam cell formation in diabetic *db/db* mice. We also demonstrated that combination therapy with SGLT2i and DPP-4i suppressed atherosclerosis in diabetic *Apoe^−/−^* mice.

Suppression of macrophage foam cell formation was comparable with ipragliflozin- and alogliptin-treated mice despite the significant difference in amelioration of hyperglycemia between the two groups. Indeed, under similar glycemic conditions, combination therapy led to increased suppression of foam cell formation compared to ipragliflozin monotherapy. Further, the extent of foam cell formation suppression was disproportionally higher in mice treated with alogliptin alone, or in combination, at corresponding HbA1c levels. Taken together, these results suggest that DPP-4i exerts a pleiotropic suppressive effect on macrophage foam cell formation beyond glucose regulation.

Our results indicate that SGLT2i therapy suppressed macrophage foam cell formation in a glucose-dependent manner. Although treatment with ipragliflozin alone, or in combination, tended to reduce body weight and SBP, these changes did not correlate with foam cell formation. There were no significant correlations between plasma lipid levels and foam cell formation. These results provide additional evidence that the glucose-lowering effect of SGLT2i therapy was solely attributable to its suppressive effect on macrophage foam cell formation. Therefore, it is highly likely that the suppressive effect of combination therapy on macrophage foam cell formation is due to both the marked amelioration of hyperglycemia by SGLT2i and the pleiotropic effect of DPP-4i.

Several studies demonstrated that high glucose upregulated the expression of scavenger receptors such as Lox-1, CD36, and scavenger receptor-A (SR-A) in cultured macrophages or dendritic cells [11–14] and that the expression of scavenger receptors in macrophages was suppressed by glucose control [11–14]. In our preliminary study, the expression of Lox-1 and CD36 were 2-3 times higher in *db/db* mice than in nondiabetic mice, but the expression of SR-A was not significantly increased in *db/db* mice compared with control mice (data not shown). Therefore, we did not examine SR-A expression in this study. The exact mechanism of hyperglycemia-induced upregulation of scavenger receptors remains unknown; one hypothesis is that hyperglycemia-mediated accumulation of reactive oxygen species induces proinflammatory signaling, including p38 mitogen-activated protein kinase (MAPK), which induces the nuclear transcription factor-kappa (NF-κ) B pathway and subsequent upregulation of scavenger receptor expression [14]. We previously reported that CD36 in mice macrophages treated with GLP-1, GIP, or DPP-4i was downregulated [5, 9]. Dai et al. demonstrated that DPP-4i reduced the expressions of Lox-1 and CD36 in macrophages [15], suggesting DPP-4i directly suppressed the macrophage foam cell formation, independent of incretins. Thus, this is the first study to demonstrate that combination therapy can remarkably suppress expression of both Lox-1 and CD36 associated with the reduction of foam cell formation. Further, our results provide evidence that both DPP-4 inhibition and glucose-lowering effect might synergistically suppress the expression of scavenger receptors.

The cholesterol efflux system plays a pivotal role in suppressing macrophage foam cell formation. Cholesterol efflux to apolipoprotein A-1 or HDL requires ABCA1 and ABCG1, respectively [8]. Accumulating experimental evidence suggest that ABCA1 or ABCG1 expression in macrophages is suppressed by high-glucose concentrations in vitro and by hyperglycemia ex vivo [16–18]. Thus, our data may provide support for the association of glycemia with ABCA1 and ABCG1 expressions. We previously reported that ABCA1 expression in human macrophages was increased by a GLP-1 receptor agonist [19]. The 3′,5′-cyclic adenosine monophosphate (cyclic-AMP)/cyclic-AMP protein kinase (PKA) pathway is a major signaling cascade of incretin receptors, and ABCA1 and ABCG1 expressions are stimulated by the activation of cyclic-AMP/PKA pathway [20, 21]. Therefore, it is possible that alogliptin treatment may stimulate the cyclic-AMP/PKA pathway in macrophages via increased endogenous incretins. Similar to results observed for the scavenger receptors, this is the first study to show that combination therapy leads to a significant increase in ABCA1 and ABCG1 expressions. Glucose-lowering action and incretin-mediated action may be both involved in enhancing the expression of key mediators of cellular cholesterol efflux.

We also found that combination therapy with SGLT2i and DPP-4i showed greatest suppression on atheromatous plaque in the aortic root. In addition, the intraplaque macrophage infiltration in the aortic root showed a similar tendency. These results indicate additive antiatherosclerotic effects of combination therapy consistent with the suppression of macrophage form cell formation observed in *db/db* mice. In contrast, combination therapy did not show any additive effect on atherosclerotic plaque formation on the surface of the aorta in *Apoe^−/−^* mice (BALB/c background). In the present study, we used *Apoe^−/−^* mice with BALB/c background that has been shown to be resistant to atherosclerosis compared with those with C56BL6 background, which are more frequently used [22]. For this reason, it is possible that atherosclerotic plaque formation was too weak to detect suppression by these agents.

## 5. Conclusions

The present study is the first to demonstrate that combination therapy with SGLT2i and DPP-4i synergistically suppresses macrophage foam cell formation in type 2 diabetic mice and atheromatous plaque formation in diabetic, dyslipidemic mice. Our findings indicate that scavenger receptors and cholesterol efflux, two major players in foam cell formation, are regulated by both glycemic control and DPP-4 inhibition.

## Figures and Tables

**Table 1:** Characteristics and laboratory data of diabetic *db/db* mice that received a normal diet containing the indicated treatments.

	Control	Ipragliflozin	Alogliptin	Combination
Number	8	8	8	7
Final body weight (g)	49.6 ± 0.4	46.5 ± 1.0	48.5 ± 0.4	46.8 ± 0.7
Food intake (g/day)	4.5 ± 0.3	4.8 ± 0.4	4.2 ± 0.2	4.9 ± 0.3
Water intake (mL/day)	4.2 ± 0.2	9.6 ± 1.0^a,c^	4.4 ± 0.3	9.9 ± 0.9^a,c^
Urine volume (mL/day)	7.6 ± 0.3	12.7 ± 1.0^a,c^	8.1 ± 0.4	13.8 ± 0.9^a,c^
SBP (mmHg)	109 ± 2	104 ± 4	107 ± 3	105 ± 2
Pulse (/min)	587 ± 36	655 ± 42	570 ± 60	672 ± 29
Glucose (mg/dL)	424 ± 37	213 ± 32^a^	231 ± 23^a^	157 ± 13^a,c^
HbA1c (%)	8.1 ± 0.1	6.7 ± 0.2^a^	7.4 ± 0.3^a,b^	6.6 ± 0.1^a,c^
Insulin (ng/mL)	5.19 ± 0.42	5.32 ± 0.89	8.20 ± 0.96^a,b^	9.56 ± 1.02^a,b^
Total-C (mg/dL)	146 ± 12	167 ± 19	147 ± 13	181 ± 13
HDL-C (mg/dL)	62 ± 5	65 ± 7	64 ± 7	86 ± 14
Triglyceride (mg/dL)	72 ± 9	61 ± 7	69 ± 13	65 ± 13
Active GLP-1 (pmol/L)	2.6 ± 0.3	2.7 ± 0.3	10.3 ± 2.2^a,b^	7.4 ± 1.1^a,b^
Total GIP (pmol/L)	49 ± 10	56 ± 14	106 ± 19^a,b^	123 ± 29^a,b^

The values show mean ± SEM. Ipragliflozin, sodium-glucose cotransporter 2 inhibitor; alogliptin, dipeptidyl peptidase-4 inhibitor; SBP, systolic blood pressure; HbA1c, hemoglobin A1c; Total-C, total cholesterol; HDL-C, high-density lipoprotein cholesterol; GLP-1, glucagon like peptide-1; GIP, glucose-dependent insulinotropic polypeptide. ^a^*p* < 0.05 versus control; ^b^*p* < 0.05 versus ipragliflozin; ^c^*p* < 0.05 versus alogliptin.

**Table 2:** Characteristics and laboratory data of streptozotocin-induced diabetic apolipoprotein E-null mice assigned to the indicated treatments.

	Control	Ipragliflozin	Alogliptin	Combination
Number	6	4	3	5
Final body weight (g)	23.7 ± 0.4	25.4 ± 0.8	25.2 ± 0.4	24.5 ± 0.4
Food intake (g/day)	4.3 ± 0.2	3.8 ± 0.2	4.5 ± 0.6	7.0 ± 0.3^a,b,c^
Water intake (mL/day)	13.7 ± 0.7	16.6 ± 1.1	13.7 ± 1.8	14.9 ± 0.7
SBP (mmHg)	122 ± 6	113 ± 5	123 ± 10	112 ± 2
Pulse (/min)	662 ± 14	675 ± 1	597 ± 6^a,b^	620 ± 13
Final body weight (g)	23.7 ± 0.4	25.4 ± 0.8	25.2 ± 0.4	24.5 ± 0.4
Glucose (mg/dL)	212 ± 33	285 ± 38	141 ± 24	192 ± 30
HbA1c (%)	8.5 ± 0.2	6.6 ± 0.3^a^	8.0 ± 0.9	6.1 ± 0.3^a^
Total-C (mg/dL)	467 ± 12	433 ± 16	475 ± 16	388 ± 33
HDL-C (mg/dL)	42 ± 4	52 ± 4	55 ± 3	47 ± 6
Triglyceride (mg/dL)	125 ± 22	161 ± 12	152 ± 22	140 ± 23

The values show mean ± SEM. ^a^*p* < 0.05 versus control; ^b^*p* < 0.05 versus ipragliflozin; ^c^*p* < 0.05 versus alogliptin.

## References

[1] Shah Z., Kampfrath T., Deiuliis J. A. (2011). Long-term dipeptidyl-peptidase 4 inhibition reduces atherosclerosis and inflammation via effects on monocyte recruitment and chemotaxis. *Circulation*.

[2] Ta N. N., Schuyler C. A., Li Y., Lopes-Virella M. F., Huang Y. (2011). DPP-4 (CD26) inhibitor alogliptin inhibits atherosclerosis in diabetic apolipoprotein E-deficient mice. *Journal of Cardiovascular Pharmacology*.

[3] Vittone F., Liberman A., Vasic D. (2012). Sitagliptin reduces plaque macrophage content and stabilises arteriosclerotic lesions in Apoe (-/-) mice. *Diabetologia*.

[4] Ervinna N., Mita T., Yasunari E. (2013). Anagliptin, a DPP-4 inhibitor, suppresses proliferation of vascular smooth muscles and monocyte inflammatory reaction and attenuates atherosclerosis in male apo E-deficient mice. *Endocrinology*.

[5] Terasaki M., Nagashima M., Watanabe T. (2012). Effects of PKF275-055, a dipeptidyl peptidase-4 inhibitor, on the development of atherosclerotic lesions in apolipoprotein E-null mice. *Metabolism*.

[6] Terasaki M., Nagashima M., Nohtomi K. (2013). Preventive effect of dipeptidyl peptidase-4 inhibitor on atherosclerosis is mainly attributable to incretin’s actions in nondiabetic and diabetic apolipoprotein E-null mice. *PLoS One*.

[7] Terasaki M., Hiromura M., Mori Y. (2015). Amelioration of hyperglycemia with a sodium-glucose cotransporter 2 inhibitor prevents macrophage-driven atherosclerosis through macrophage foam cell formation suppression in type 1 and type 2 diabetic mice. *PLoS One*.

[8] Allahverdian S., Pannu P. S., Francis G. A. (2012). Contribution of monocyte-derived macrophages and smooth muscle cells to arterial foam cell formation. *Cardiovascular Research*.

[9] Nagashima M., Watanabe T., Terasaki M. (2011). Native incretins prevent the development of atherosclerotic lesions in apolipoprotein E knockout mice. *Diabetologia*.

[10] Kohashi K., Hiromura M., Mori Y. (2016). A dipeptidyl peptidase-4 inhibitor but not incretins suppresses abdominal aortic aneurysms in angiotensin II-infused apolipoprotein E-null mice. *Journal of Atherosclerosis and Thrombosis*.

[11] Li L., Sawamura T., Renier G. (2004). Glucose enhances human macrophage LOX-1 expression: role for LOX-1 in glucose-induced macrophage foam cell formation. *Circulation Research*.

[12] Griffin E., Re A., Hamel N. (2001). A link between diabetes and atherosclerosis: glucose regulates expression of CD36 at the level of translation. *Nature Medicine*.

[13] Fukuhara-Takaki K., Sakai M., Sakamoto Y., Takeya M., Horiuchi S. (2005). Expression of class a scavenger receptor is enhanced by high glucose in vitro and under diabetic conditions in vivo: one mechanism for an increased rate of atherosclerosis in diabetes. *The Journal of Biological Chemistry*.

[14] Lu H., Yao K., Huang D. (2013). High glucose induces upregulation of scavenger receptors and promotes maturation of dendritic cells. *Cardiovascular Diabetology*.

[15] Dai Y., Wang X., Ding Z., Dai D., Mehta J. L. (2014). DPP-4 inhibitors repress foam cell formation by inhibiting scavenger receptors through protein kinase C pathway. *Acta Dibetologica*.

[16] Chang Y. C., Sheu W. H., Chien Y. S., Tseng P. C., Lee W. J., Chiang A. N. (2013). Hyperglycemia accelerates ATP-binding cassette transporter A1 degradation via an ERK-dependent pathway in macrophages. *Journal of Cellular Biochemistry*.

[17] Mauerer R., Ebert S., Langmann T. (2009). High glucose, unsaturated and saturated fatty acids differentially regulate expression of ATP-binding cassette transporters ABCA1 and ABCG1 in human macrophages. *Experimental & Molecular Medicine*.

[18] Machado-Lima A., Iborra R. T., Pinto R. S. (2015). In type 2 diabetes mellitus glycated albumin alters macrophage gene expression impairing ABCA1-mediated cholesterol efflux. *Journal of Cellular Physiology*.

[19] Tashiro Y., Sato K., Watanabe T. (2014). A glucagon-like peptide-1 analog liraglutide suppresses macrophage foam cell formation and atherosclerosis. *Peptides*.

[20] Nishiuchi Y., Murao K., Imachi H., Nishiuchi T., Iwama H., Ishida T. (2010). Transcriptional factor prolactin regulatory element-binding protein-mediated gene transcription of ABCA1 via 3′,5′-cyclic adenosine-5′-monophosphate. *Atherosclerosis*.

[21] Gelissen I. C., Sharpe L. J., Sandoval C. (2012). Protein kinase A modulates the activity of a major human isoform of ABCG1. *Journal of Lipid Research*.

[22] Schulte S., Sukhova G. K., Libby P. (2008). Genetically programmed biases in Th1 and Th2 immune responses modulate Atherogenesis. *The American Journal of Pathology*.

